# The Changes, Aggregation Processes, and Driving Factors for Soil Fungal Communities during Tropical Forest Restoration

**DOI:** 10.3390/jof10010027

**Published:** 2023-12-31

**Authors:** Xiaofei Guo, Shaojun Wang, Chen Wang, Mengjie Lan, Shengqiu Yang, Shuang Luo, Rui Li, Jiahui Xia, Bo Xiao, Lingling Xie, Zhengjun Wang, Zhipeng Guo

**Affiliations:** 1 College of Ecology and Environment, Southwest Forestry University, Kunming 650224, China; guoxiaofeins@163.com (X.G.);; 2 College of Water Conservancy, Yunnan Agricultural University, Kunming 650201, China

**Keywords:** assembly processes, deterministic process, fungal assemblages, tropical forest restoration, Xishuangbanna

## Abstract

Soil fungal communities play crucial roles in mediating the functional associations between above- and belowground components during forest restoration. Forest restoration shapes the alterations in plant and soil environments, which exerts a crucial effect on soil fungal assemblages. However, the changes, assembly processes, and driving factors of soil fungi communities during tropical forest restoration are still uncertain. We used Illumina high-throughput sequencing to identify the changes of soil fungal communities across a tropical secondary forest succession chronosequence (i.e., 12-, 42-, and 53-yr stages) in Xishuangbanna. During forest restoration, the dominant taxa of soil fungi communities shifted from r- to K-strategists. The relative abundance of Ascomycota (r-strategists) decreased by 10.0% and that of Basidiomycota (K-strategists) increased by 4.9% at the 53-yr restoration stage compared with the 12-yr stage. From the 12-yr to 53-yr stage, the operational taxonomic unit (OTU), abundance-based coverage estimator (ACE), Chao1, and Shannon index of fungal communities declined by 14.5–57.4%. Although the stochastic processes were relatively important in determining fungal assemblages at the late stage, the fungal community assembly was dominated by deterministic processes rather than stochastic processes. The shifts in soil properties resulting from tropical forest restoration exerted significant effects on fungal composition and diversity. The positive effects of microbial biomass carbon, readily oxidizable carbon, and soil water content explained 11.5%, 9.6%, and 9.1% of the variations in fungal community composition, respectively. In contrast, microbial biomass carbon (40.0%), readily oxidizable carbon (14.0%), and total nitrogen (13.6%) negatively contributed to the variations in fungal community diversity. Our data suggested that the changes in fungal composition and diversity during tropical forest restoration were primarily mediated by the positive or negative impacts of soil carbon and nitrogen pools.

## 1. Introduction

As an important functional component in the forest ecosystem, soil fungi play a vital role in regulating organic matter decomposition [1], nutrient cycling [2], plant diversity, and ecosystem productivity [3,4]. Soil fungal assemblages can be mediated by the alterations in soil and plant characteristics during vegetation community succession [5,6,7,8]. In the process of forest restoration, the increasing colonization of plants and the deepening of root penetration into the soil may elevate vegetation coverage and soil porosity, thereby improving the habitat conditions of the fungi [9,10]. The inputs of litter biomass and root exudates increase with the aging of the forest and the elevation of tree diversity, which can meet the increasing nutrient needs of soil fungi during forest restoration [7,10]. These effects of forest community succession could play a critical role in shaping the shifts in soil fungal composition and diversity [6,11,12]. A lot of studies confirmed the responses of soil fungal communities to vegetation succession among ecosystem types such as boreal lands [11,13], peatlands [14], alpine grasslands [12,15], desert ecosystems [16,17], temperate forests [18,19], and subtropical forests [5,20]. However, there is no consistent conclusion about the dynamics of soil fungal communities during tropical forest succession [21,22]. Therefore, it is critical to determine the mechanisms by which tropical forest succession can regulate the composition and diversity of soil fungi communities.

Soil fungal communities are composed of taxa with different nutritional strategies. Some fungal groups belong to r-strategists (copiotrophic species) that allocate more energy to reproduction than to growth, metabolism, and competitiveness; thus, they are more adaptive to unstable ecosystems enriched in labile nutrients [23,24]. Mature ecosystems with more favorable environment conditions encourage the r-strategists to give way to the K-strategists (oligotrophic groups) with lower reproductive rates, higher survival rates, and greater efficiency in using stable substrates [23,25]. However, some studies observed that forest vegetation restoration changed microbial communities from oligotrophic groups to copiotrophic groups [26,27]. Moreover, restoration-driven plant richness and diversity may support higher fungal diversity through providing various qualities of litter, root exudates, microenvironment, and symbiotic hosts [28,29]; while increased soil nutrients could meet the different metabolic needs of various fungal function guilds [30,31,32]. However, the decline in fungal diversity during with forest recovery has also been reported [33]. This means that there are a lot of uncertainties in determining the linkage of soil microbial communities with vegetation restoration. Thus, it is pivotal to identify the changes in soil fungal composition and diversity during forest restoration.

Stochastic and deterministic processes are two types of ecological determinations that control the assemblages in soil microbial communities [34]. Stochastic processes involve probabilistic dispersal and ecological drift that lead to random changes in microbial community compositions [35]. In contrast, deterministic processes emphasize the driving role of environmental filtering and niche partitioning [36,37]. Previous studies have suggested that the relative importance of stochastic and deterministic processes in structuring microbial community assembly varies with vegetation restoration [34,38]. An increase in plant species abundance and diversity affects carbon (C) inputs to the soil by altering litter inputs and root exudates, thereby altering the assembly process of C-utilizing microbial community [34,39,40]. Furthermore, increased availability of soil resources may reduce the importance of deterministic processes [41]. Greater stochasticity has been observed in determining the assemblages in fungal communities at high nutrient concentration agro-soils [42]. The relative importance of deterministic and stochastic processes in governing fungal assemblages during tropical forest restoration, however, is still uncertain. Therefore, it is critical to determine the relative importance of stochastic and deterministic processes in driving the alterations in soil fungal communities during tropical forest restoration [34,40].

The deterministic mechanism by which forest restoration affects soil fungi is primarily focused on the feedback relationships between above- and belowground components [43,44,45]. Soil properties are key regulators that link above plant performance with below microbial communities during forest restoration [10,46]. Previous studies have shown that restoration-driven changes in soil pH, moisture, and nutrient availability significantly affect microbial communities [47,48]. However, the effects of respective soil variables on fungal communities appear to be inconsistent across different ecosystems. For example, some studies observed significant effects of soil pH on fungal composition and diversity [49,50], while others found no significant effect on fungal community [51]. Soil nitrogen (N) availability is crucial to determining fungal diversity [52], which is inconsistent with the findings that nitrogen addition had no significant effect on fungal diversity [53]. Therefore, it is critical to clarify the contribution of respective soil variables to composition and the diversity of fungal community during forest restoration.

Xishuangbanna is the concentrated distribution area of tropical rainforests in China. These tropical forests, however, were severely damaged before the ceasing of slash–burn agricultural practices. A sequence of restoration stages has thus been generated during secondary forest succession, which would provide distinguished research sites to link the associations of soil properties with fungal communities during tropical forest restoration. We aimed to (i) explore the dynamics of fungal composition and diversity during tropical forest restoration; (ii) determine whether deterministic or stochastic processes dominate fungal community assembly; and (iii) identify the major soil variables in determining changes in fungal community during tropical forest restoration. We hypothesized that (i) tropical forest restoration can positively affect fungal composition and diversity via its modification on soil physical and nutrient environments; (ii) deterministic processes might be more dominant than stochastic processes because restoration-induced changes in plant and soil environments could impose a selected effect on fungal community development; and (iii) soil physical environment and nutrient pools would play different roles in regulating fungal community changes.

## 2. Materials and Methods

### 2.1. Experiment Designs

The sites were located in the Xishuangbanna Tropical Botanical Garden (21°55′ N, 101°15′ E) in southern Yunnan of China, where the annual average temperature and rainfall are approximately 21.5 °C and 1557 mm, respectively. About 87% of the rainfall occurs during the wet season (from May to October). Soil type is classified as Oxisols. The air and soil are at a high humidity due to the heavy fog from night to daytime, resulting in the prosperity of tropical forests in this area. But the primary tropical forests had been destroyed by the slash-and-burn farming practices until the 1960s, when a protection program was implemented to cease the destruction. Accordingly, a series of secondary forest stages have been developed.

The experiments were conducted in an age-chronosequence of restoration stages (i.e., 12-yr *Mallotus paniculatus* community, 42-yr *Mellettia leptobotrya* community, and 53-yr *Syzygium oblatum* community) to investigate the effects of tropical forest restoration on shifts in fungal communities in 2018. The three restoration stages were derived from the lands which were abandoned after slash-and-burn agriculture in 2006, 1976, and 1965, respectively. Each stage was located 200–1000 m apart. Five repeated sites (50 × 40 m, 200 m apart) at each stage and three reduplicative plots (15 × 10 m, 10 m apart) within each site were randomly set up. Thus, a total of 15 plots were sampled at each restoration stage. The similarity of natural conditions (e.g., soil type and microtopography) and human interventions (e.g., burning degree of slash-and-burn and management history) at each site guaranteed the condition similarity within these different stages.

### 2.2. Soil Physicochemical Analysis

Soil samples were collected at each plot of three restorations in November 2018. Per plot, three soil cores (Ø 5 cm × 10 cm deep, 4 m apart) were taken to measure bulk density (BD). Three replicated soil samples were randomly collected at 0–10 cm soil depth at each plot and then fully mixed into one composite sample. Each mixed soil sample was divided into four parts. One part was used to determine soil water content (SW), drying 20 g of fresh soil at 105 °C for 24 h. Another part was air-dried and then sieved with a 2.0 mm sifter to analyze soil pH, soil organic carbon (SOC), readily oxidizable carbon (ROC), total nitrogen (TN), and hydrolysable nitrogen (HN). The remaining two parts were transported on ice to the laboratory for the determination of microbial biomass carbon (MBC), NH_4_–N, and NO_3_–N and the extraction of DNA immediately; otherwise, they would be stored at 4 °C and −80 °C, respectively. MBC was determined using chloroform fumigation extraction [54]. Soil pH was measured utilizing a glass electrode in solutions (g ml^−1^) of 1:2.5 soil:water. SOC and TN were analyzed through the H_2_SO_4_-K_2_Cr_2_O_7_ method and the Kjeldahl method, respectively. ROC was assayed by a KMnO_4_ (333 mM) oxidation procedure [55]. HN was determined by the alkaline hydrolysis diffusion method. The concentrations of NO_3_–N and NH_4_–N were gauged with a UV-VIS spectrophotometer (UV mini 1240, Shimadzu, Kyoto, Japan).

### 2.3. DNA Extraction, Amplification, Sequencing Data Processing

The soil genomic DNA was extracted from 0.5 g fresh soil using the E.Z.N.A. DNA Kit (Omega Bio-tek, Norcross, GA, USA) following the manufacturer’s instructions. The extracted DNAs were checked on 1% agarose gels, using a NanoDrop 2000 spectrometer (Thermo Fisher Scientific Inc., MA, USA). The fungal internal transcribed spacer 1 (ITS1) region was amplified by PCR using the primers ITS1F (5′-CTTGGTCATTTAGAGGAAGTAA-3′) [56] and ITS2R (5′-GCTGCGTTCTTCATCGATGC-3′) [57] as follows: pre-denaturation at 95 °C for 3 min, followed by 35 cycles of denaturing at 95 °C for 30 s, annealing at 55 °C for 30 s, and extension at 72 °C for 45 s, a single extension at 72 °C for 10 min, and ending at 10 °C. PCR reactions were conducted in triplicate using 20 μL mixture containing 4 μL of 5× FastPfu Buffer, 2 μL of 2.5 mM dNTPs, 0.8 μL of each primer (5 μM), 0.4 μL of FastPfu Polymerase, 0.2 μL of BSA, 10 ng of template DNA, and ddH_2_O to replenish the remaining volume. The resulting amplicons were extracted from a 2% agarose gel and then further purified using the AxyPrep DNA Gel Extraction Kit (Axygen Biosciences, Union City, CA, USA). Quantification of purified PCR products were performed using Qubit^®^3.0 (Life Invitrogen, Thermo Fisher Scientific Inc., MA, USA), and every twenty-four amplicons with different barcodes were mixed equally. Following Illumina’s genomic DNA library preparation procedure, the pooled DNA products were utilized to construct Illumina Pair-End library. Then, the amplicon library was paired-end sequenced (2 × 300) on an Illumina MiSeq platform (Shanghai BIOZERON Co., Ltd., Shanghai, China) according to the standard protocols.

Demultiplexing and quality-filtering of raw fastq files were conducted using QIIME2 (version 2018.8) with the following criteria: (i) the raw sequence reads that were <50 bp, had an average quality score < 20, or contained ambiguous bases were screened out; (ii) sequences with barcode mismatches > 0 or base mismatches > 2 were removed; and (iii) only sequences with overlap > 10 bp and mismatches < 0.2 would be assembled according to their overlap sequence. High-quality sequences were assigned to the same operational taxonomic units (OTUs) at 97% similarity threshold using Usearch (version 10 http://drive5.com/uparse/, accessed on 13 December 2018) and chimeric sequences were removed using UCHIME. Taxonomy was assigned to each OTU by the RDP classifier trained on the UNITE database. Additionally, the sequence of each unclassified OTU was individually conducted via a BLASTn search against the National Center for Biotechnology Information (NCBI) GenBank database to determine their origin. The choice of the closest match was determined by rankings based on e-value, Query coverage scores, and the ident% [58]. The OTU would be discarded once the highest ranked match was non-fungal. Otherwise, the OTU would be identified as a specific or unclassified fungus based on whether the best-matched sequence had a clear taxonomic affiliation.

### 2.4. Data Analysis

The more significant models of regression analysis were selected to fit the variation tendency of SW, BD, pH, SOC, ROC, MBC, TN, HN, NH_4_–N, and NO_3_–N during tropical forest restoration via SigmaPlot 15.0 software. To compare fungal communities at three restoration stages at the same sequencing depth, subsequent analyses were performed based on data obtained by randomly sampling the sequences of each sample to the minimum sequence size (27,874) among all samples using the vegan package. The OTU, ACE, Chao1, and Shannon diversity were calculated for soil fungi communities. Fisher’s one-way analysis of variance (ANOVA) was employed to estimate the effect of forest restoration stages on fungi composition and diversity if these variables can pass the normality and the homogeneity tests for variance. Tukey’ honest significant difference (HSD) was used to examine the differences among three restoration stages (*p* < 0.05). Non-metric multidimensional scaling (NMDS) (Bray–Curtis distance algorithm) was performed to analyze the beta diversity of soil fungi across the three restoration stages using vegan package. Simultaneously, analysis of similarity (ANOSIM) was utilized to identify the differences in the fungal community compositions among the three restoration stages. Null model-based normalized stochastic ratios (NST) were calculated with Jaccard to assess the influences of deterministic and stochastic processes on fungal community assembly. The redundancy analysis (RDA) was performed to estimate which soil properties best explained changes in fungal community. Forward selection with Monte Carlo permutation (999 repetitions) was performed to choose the soil properties that significantly contributed to fungal community changes by using the ‘forward. sel’ function of the adespatial package. All analyses were implemented using R software (version 4.2.2) except when indicated otherwise.

## 3. Results

### 3.1. Changes in Fungal Diversity and Structure during Tropical Forest Restoration

**Figure 1:**
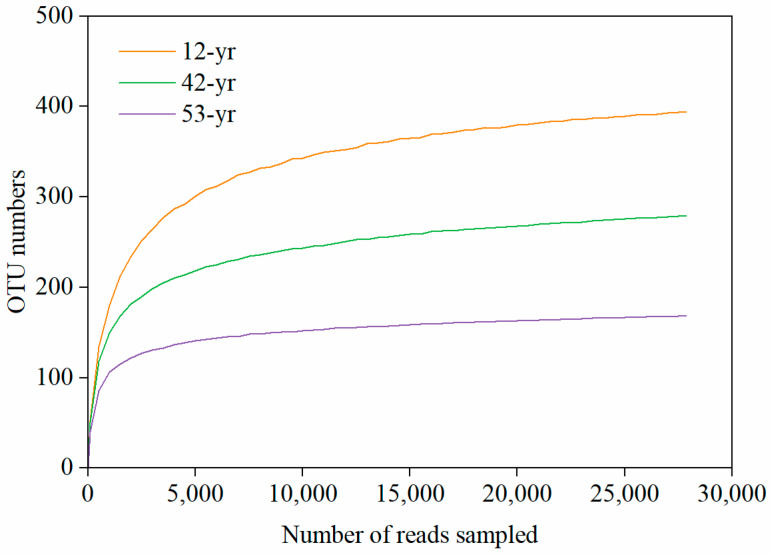
Multi-sample rarefaction curves of high-throughput sequencing for soil fungi during tropical forest restoration.

**Figure 2:**
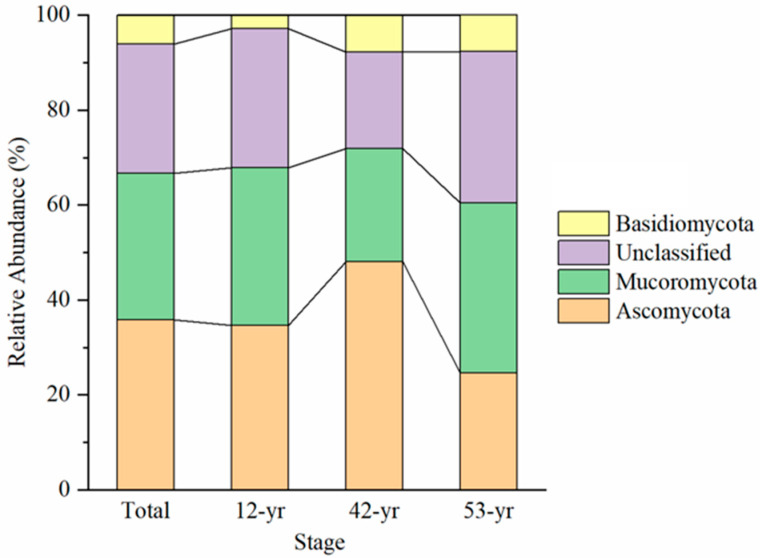
The alteration of soil fungal composition at phylum level during tropical forest restoration.

The rarefaction curves leveled off with the increase in sequencing data, which indicated that the majority of the soil fungi were covered by the obtained sequences at 97% similarity level (Figure 1). A total of 418,110 sequences obtained were assigned to five known fungal phyla. The groups of Ascomycota (35.8%) and Mucoromycota (31.0%) were the two dominant phyla, followed by Basidiomycota (6.0%) and others (<0.1%), which contained Chytridiomycota and Blastocladiomycota (Figure 2). Nevertheless, there were still more than 27.2% of the sequences of unclassified taxa because they could not be matched with any known fungal phylum (Figure 2). The relative abundance of Ascomycota (48.1%) was higher at the 42-yr restoration stage than at the 12- and 53-yr restoration stages (Figure 2). In contrast, the relative abundances of Mucoromycota and unclassified groups were lower at 42-yr stage (Figure 2). The Basidiomycota had the lowest relative abundance at the 12-yr restoration stage (Figure 2).

**Figure 3:**
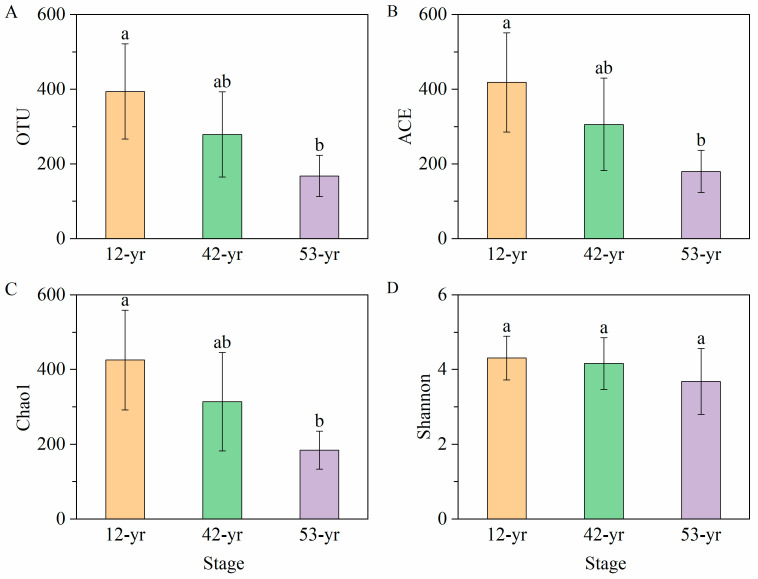
The changes in OTU (**A**), ACE (**B**), Chao1 (**C**), and Shannon (**D**) during tropical forest restoration in Xishuangbanna. Columns denote mean ± SD (standard deviation). Different lowercase letters are significantly different (*p* < 0.05) among three restoration stages (ANOVA with Tukey’s honest significant difference (HSD)).

We observed significant changes in the OTU, ACE and Chao1 diversity of soil fungal communities during tropical forest restoration. The OTU richness decreased by 19.7–57.4% during the forest restoration (Figure 3A, *p* < 0.05), while ACE and Chao1 in the 53-yr stage decreased by 57.0% and 56.6%, respectively, compared with the 12-yr stage (Figure 3B,C, *p* < 0.05). There was no significant decrease in the Shannon index during forest restoration (Figure 3D, *p* > 0.05).

**Figure 4:**
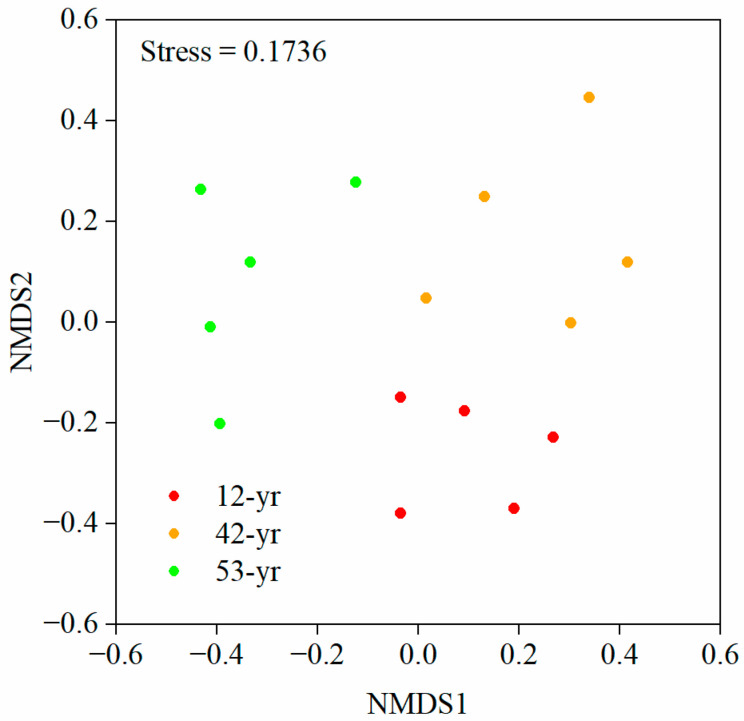
Non-metric multidimensional scaling (NMDS) of soil fungal community structure at different restoration stages.

For beta diversity, the results of NMDS showed that fungal operational taxonomic units (OTUs) formed three clearly distinguishable clusters during tropical forest restoration (Figure 4). Furthermore, according to the criteria described by Zhou et al. [59], there were significant differences in the fungal community structure throughout the forest restoration stages, although that of 42-yr vs. 53-yr stage was separated but strongly overlapped (Table 1).

### 3.2. Deterministic and Stochastic Processes of Soil Fungal Community Assembly

**Figure 5:**
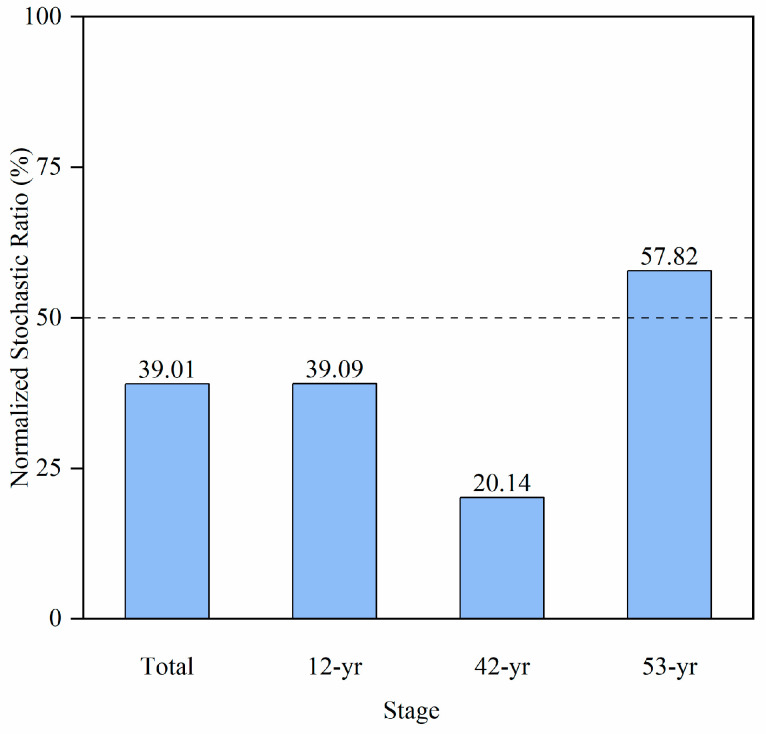
The normalized ratios of stochastic process in controlling fungi community change across tropical forest restoration based on the Jaccard indexes.

The normalized stochastic ratios contributed 39.01% to alteration of soil fungi communities based on all samples (Figure 5). The NST at 53-yr restoration stage (57.82%) was higher than those at 12-yr (39.09%) and 42-yr (20.14%) restoration stages. In particular, the 42-yr stage had the lowest NST compared with other two restoration stages. According to the criteria of the boundary point (50%) for determining whether deterministic processes (<50%) or stochastic processes (>50%) are more dominant [60], we found that deterministic processes made the main contribution to fungal community assembly in the 12- and 42-yr restoration stages, while stochastic processes were prevailing in the 53-yr restoration stages.

### 3.3. Shifts in Soil Properties during Tropical Forest Restoration

**Figure 6:**
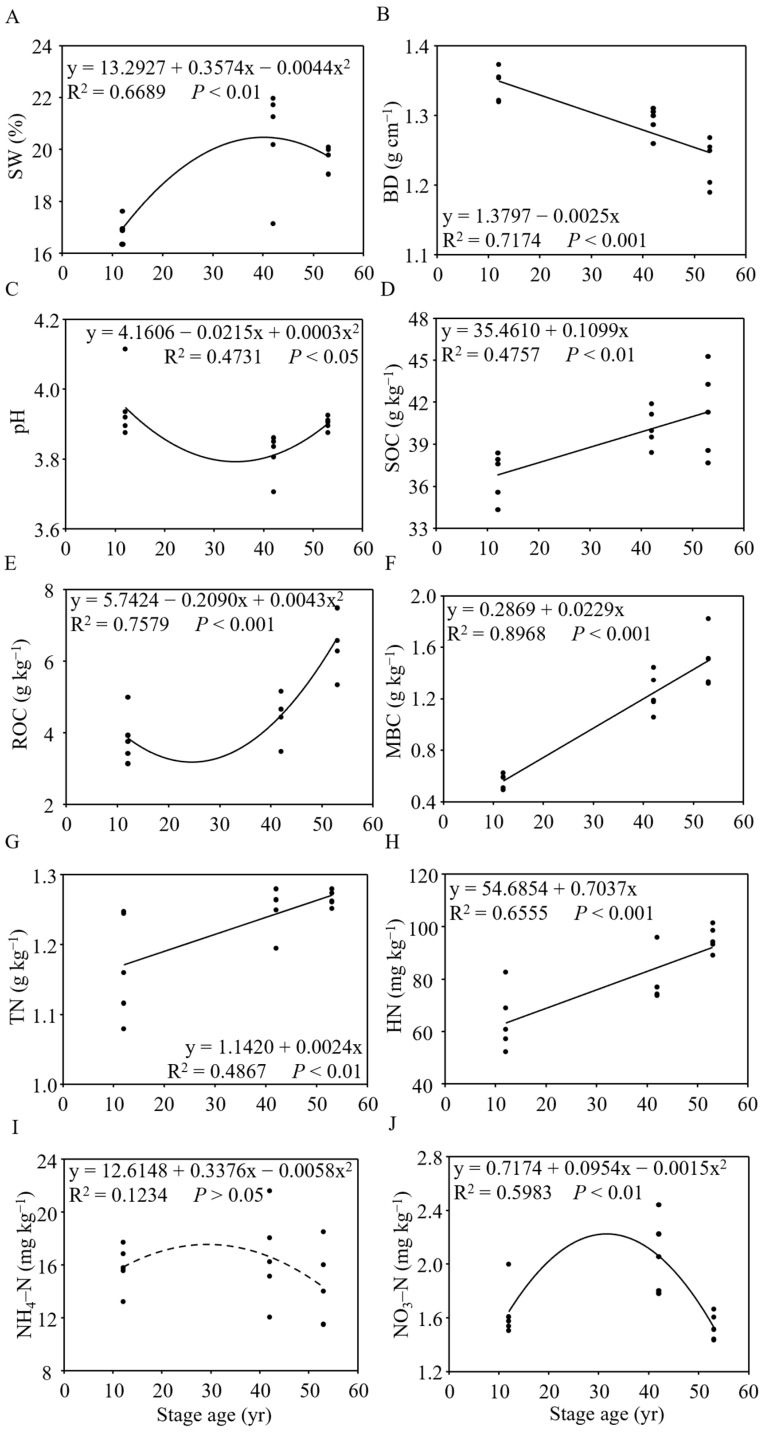
Changes in SW (**A**), BD (**B**), pH (**C**), SOC (**D**), ROC (**E**), MBC (**F**), TN (**G**), HN (**H**), NH_4_–N (**I**), and NO_3_–N (**J**) in response to tropical forest restoration. The solid and dashed lines indicate the presence and absence of significant relationships. SW, soil water content; BD, bulk density; pH, pH; SOC, soil organic carbon; MBC, microbial biomass carbon; ROC, readily oxidizable carbon; TN, total nitrogen; HN, hydrolysable nitrogen; NH_4_–N, NH_4_–N; NO_3_–N, NO_3_–N.

We observed that all measured soil properties varied significantly across three forest restoration stages, except for NH_4_–N (Figure 6). Compared to the 12-yr restoration stage, SW increased to 1.21-fold in the 42-yr stage and 1.17-fold in 53-yr stage, respectively, while BD decreased by 3.9–4.6% during the forest restoration. The soil pH initially decreased (3.5%) and then increased (2.4%) during tropical forest restoration. Soil carbon pools (i.e., SOC, ROC, and MBC) increased by 1.12–2.67 fold during forest restoration from the 12-yr stage to the 53-yr stage. Furthermore, the soil nitrogen pools (i.e., TN, HN, NH_4_–N, and NO_3_–N) had different variations during forest restoration. The values of TN and HN increased to 1.08-fold and 1.48-fold, respectively, following forest restoration. The NO_3_–N initially increased (25.4%) and then decreased (25.6%) during tropical forest restoration. There was no significant change in NH_4_–N during forest restoration.

### 3.4. Linking Fungal Communities to Soil Properties

**Figure 7:**
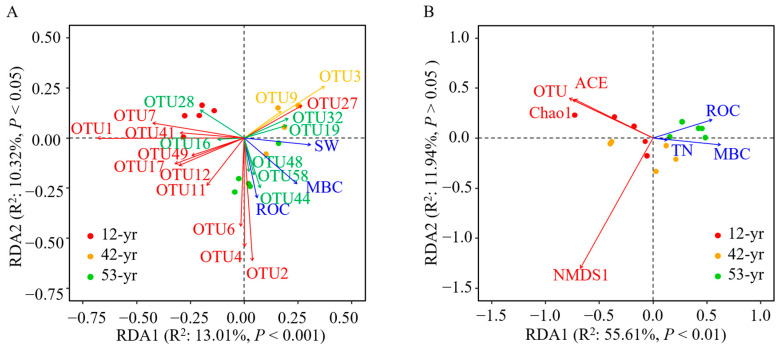
Redundancy analysis (RDA) of the linkages of fungal composition (**A**) and diversity (**B**) with soil properties. Only the top 20 OTUs in fungi composition are shown. The red, orange, and green OTUs are classified as Mucoromycota, Ascomycota, and unclassified taxa, respectively. Only significant (*p* < 0.05) factors including SW, MBC, ROC, and TN are shown. SW, soil water content; MBC, microbial biomass carbon; ROC, readily oxidizable carbon; TN, total nitrogen.

We observed that the MBC (*p* < 0.01), ROC (*p* < 0.01), and SW (*p* < 0.05) contributed 11.5%, 9.6%, and 9.1%, respectively, to the variations in OTUs of fungal taxa (Table 2 and Figure 7A). The relative abundances of the top 20 OTUs had different relationships with the three variables (Figure 7A). The Mucoromycota (OTU2, 4, and 6) and unclassified taxa (OTU44, 48, and 58) had positive linkages with ROC. In contrast, Mucoromycota (OTU1, 7, and 41) and unclassified groups (OTU16, and 28) negatively correlated with MBC and SW. In addition, Ascomycota (OTU3 and 9), Mucoromycota (OTU27), and unclassified (OTU19 and 32) groups had a positive association with SW.

We also found that MBC (*p* < 0.01), ROC (*p* < 0.05), and TN (*p* < 0.05) were the significant variables that explained 40.0%, 14.0%, and 13.6%, respectively, of the variations in fungal diversity (Table 2 and Figure 7B). Soil alpha diversity (OTU, ACE, and Chao1) had a closely negative association with MBC and TN (Figure 7B), while the beta diversity (NMDS1) was closely negatively correlated with ROC (Figure 7B).

## 4. Discussion

### 4.1. Effect of Tropical Forest Restoration on Fungi Community Structure

The present study observed that the dominant taxa in the soil fungal community composition shifted from r- to K-strategists during tropical forest development. The relative abundance of the dominant phylum (Ascomycota) initially increased and then decreased during tropical forest restoration, which was similar with the results from Liu et al. [33]. The copiotrophic Ascomycota (r-strategists) have been documented to be able to survive in barren soil conditions at the early succession stages [24,61,62]. Previous study has shown that the dominant phylum (Ascomycota) are pathogens of plants, arthropods, and animals [63]. They are very adaptable to environmental changes and have a high rate of gene replacement [64]. As a result, the Ascomycota group prevailed over oligotrophic groups in the early restoration stage.

During tropical forest restoration, the relative abundance of K-strategy fungi (Basidiomycota) increased due to increased litter input and soil nutrient availabilities. The increase in Basidiomycota abundance may be stimulated by the increasing litter input during forest restoration [65,66]. In particular, Basidiomycota are considered to be the most capable microbial group in breaking down the refractory compound (such as lignin), because of their capability to produce enzymes to decompose complex polymers cumulation of recalcitrant organic matter that may increase during forest restoration [1,66,67,68]. Therefore, the increase in plant litter input could elevate the abundance of K-strategy fungi (Basidiomycota) during tropical forest restoration.

### 4.2. Effect of Tropical Forest Restoration on Fungi Community Diversity

We did not confirm the hypothesis about the increased alpha diversity of soil fungi with tropical forest restoration. This may be attributed to the fact that forest restoration leads to a more competitive environment with fewer niche opportunities, resulting in reduction of microbial taxa [27,69,70]. In the present study, OTU, ACE, and Chao1 decreased, which is consistent with the decline trend in fungi diversity during the secondary succession of subtropical and temperate forests [5,33,71]. Some studies have found that there is higher fungal diversity and more specialized eukaryotes in the early stages than in the late stages [70,72]. This is due to the fact that a disturbed ecosystem may increase niche availability, allowing for competitive release at local scales [71,72]. For this study, some rare taxa (Chytridiomycota and Blastocladiomycota) presented at the 12-yr and 42-yr restoration stages, but they were absent at the 53-yr restoration stage. In contrast, the relative abundance of competitive Basidiomycota (K-strategists) increased during forest restoration. These results suggest that the decline in fungal diversity may have resulted from the niche loss of rare species and the increase in competitive K-strategists during tropical forest development.

### 4.3. Effect of Stochastic and Deterministic Processes on Fungi Assemblages

This study showed that soil fungal community assembly was generally governed by deterministic processes, which supported our second prediction. This may be due to the selection pressure of restoration-induced changes in plant composition, the micro-environment, and nutrient availability in soil fungi communities [38]. However, stochastic processes prevailed over deterministic processes in the late restoration stage, which is in line with previous findings [34]. This suggests that the relative effects of deterministic and stochastic processes on fungal community assembly varied across different forest restoration stages. In the early restoration stages, the lower concentration of soil nutrients may exert selection pressure on soil fungi development. The fungi need to adapt and survive in heterogeneous environments that have been restored from previously disturbed slash-and-burn agricultural land. Thus, deterministic processes dominated fungal community assembly in the early stages. In the late restoration stage, the weak environmental selection resulting from increased environmental homogeneity reduced the importance of deterministic processes [33,34,73]. In particular, increased resource supply due to litter input and root secretion not only reduced niche selection but also enhanced the effects of stochasticity [74]. Therefore, the dominance of deterministic processes was overwhelmed by stochastic processes in the later stage.

### 4.4. Effect of Restoration-Driven Alterations of Soil Variables on Fungi Assemblages

We observed the main contributions of soil variables (i.e., MBC, ROC, and SW) to the changes in soil fungal community composition during tropical forest restoration (Figure 7A). In this study, OTUs belonging to dominant phylum Mucoromycota (>99%) were classified as *Mortierella*. Previous studies have confirmed that the relative abundance of *Mortierella* was closely related to carbon sequestration and nutrient availability in soils [75,76]. The common lifestyle of *Mortierella* is regarded as soil-inhabiting saprobic organisms on decaying organic matter [77]. They have shown great capacity to degrade plant litter and refractory organic carbon into unstable carbon [78,79]. This is consistent with the observed associations between soil carbon components (MBC and ROC) and fungal composition (Figure 7A). In addition, soil moisture had a crucial effect on the abundance of fungal groups, which may be associated with the transport and diffusion of soil nutrients that are directly and indirectly mediated by soil moisture [80,81].

This study observed a negative relationship between soil fungal diversity and nutrient availability (MBC, SOC, and TN). This is discrepant with the results reported by Kim et al. [2] and Xu et al. [82], who observed positive effects of soil carbon and nitrogen on fungal alpha diversity. This contradiction might be due to the possibility that the positive or negative correlation between such soil nutrients and fungal diversity may be mediated by bacteria. Interactions between fungi and bacteria are common in soils [25,83,84]. Competition and exclusion in the development of the bacterial community due to the increased soil nutrient availability might lead to the partial niche loss of soil fungi. Bastian et al. [24] have pointed out that some bacteria (e.g., *Streptomyces*, *Bacillus*, *Cytophaga*, and *Micromonospora*) have the function of decomposing cellulose. They may have a similar function with respect to the dominant fungal phyla (Ascomycota and Basidiomycota). This could result in substrate competition between fungi and bacteria due to shared similar resources. In addition, bacteria can produce antifungal metabolites and inhibit spore germination [85,86,87], which might affect the development of fungal community. In particular, Wang et al. [27] have observed a positive development in bacterial diversity with the increased C and N stoichiometry in the litter–soil–microbe continuum during tropical forest restoration, which might support our speculation about the effect of bacteria on fungi.

## 5. Conclusions

This study observed significant changes in soil fungal composition and diversity during tropical forest restoration. The dominant taxa in soil fungal communities shifted from r- to K-strategists and fungal diversity generally decreased during tropical forest restoration. The copiotrophic Ascomycota (r-strategists) increased in the early restoration stage and decreased in the late stage. In contrast, oligotrophic Basidiomycota (K-strategists) increased first and then stabilized during tropical forest restoration. Soil fungal community assemblages were dominated by deterministic processes in the early stages, while they were primarily determined by stochastic processes in the later stage. The changes in fungal composition were positively associated with microbial biomass carbon, readily oxidizable carbon, and soil water content. In comparison, fungal community diversity was negatively correlated with soil microbial biomass carbon, readily oxidizable carbon, and total nitrogen. Our results suggest that soil carbon pool, total nitrogen, and water conditions are important deterministic factors affecting the changes in fungal composition and diversity during tropical forest restoration.

## Figures and Tables

**Table 1:** Analysis of similarity (ANOSIM) of soil fungal community structure at different restoration stages. Significant levels: *** *p* < 0.001, ** *p* < 0.01, * *p* < 0.05.

Stage	R	*p*	Significance	Description
12-yr vs. 42-yr	0.61	0.008	**	significantly separated but overlapped
12-yr vs. 53-yr	0.52	0.008	**	significantly separated but overlapped
42-yr vs. 53-yr	0.47	0.020	*	separated but strongly overlapped
overall	0.50	0.001	***	significantly separated but overlapped

**Table 2:** Variations in fungal composition and diversity explained by the most important soil variables (RDA using forward selection with Monte Carlo permutation, 999 repetitions). SW, soil water content; MBC, microbial biomass carbon; ROC, readily oxidizable carbon; TN, total nitrogen.

Variations	Variables	MBC	ROC	TN	SW	Residuals	Total
Fungal composition	R^2^	0.115	0.096	-	0.091	0.698	1.000
	R^2^ (Cumulative)	0.115	0.211	-	0.302	1.000	1.000
	*p*	0.003	0.010	-	0.027	-	-
Fungal diversity	R^2^	0.400	0.140	0.136	-	0.324	1.000
	R^2^ (Cumulative)	0.400	0.540	0.676	-	1.000	1.000
	*p*	0.003	0.043	0.022	-	-	-

## Data Availability

The raw data are available in the NCBI Sequence Read Archive (SRA) under the BioProject PRJNA1049749.
